# Beyond Fixed Thresholds: Cluster-Derived MRI Boundaries Improve Assessment of Crohn’s Disease Activity

**DOI:** 10.3390/jcm14217523

**Published:** 2025-10-23

**Authors:** Jelena Pilipovic Grubor, Sanja Stojanovic, Dijana Niciforovic, Marijana Basta Nikolic, Zoran D. Jelicic, Mirna N. Radovic, Jelena Ostojic

**Affiliations:** 1 Center for Radiology, University Clinical Center of Vojvodina, 21000 Novi Sad, Serbia; jelenapilipovicgrubor@uns.ac.rs (J.P.G.); sanja.stojanovic@mf.uns.ac.rs (S.S.); dijana.niciforovic@mf.uns.ac.rs (D.N.); marijana.basta-nikolic@mf.uns.ac.rs (M.B.N.); 2 Faculty of Medicine, University of Novi Sad, 21000 Novi Sad, Serbia; 3 Department of Computing and Control, Faculty of Technical Sciences, University of Novi Sad, 21000 Novi Sad, Serbia; jelicic@uns.ac.rs (Z.D.J.); mirna.kapetina@uns.ac.rs (M.N.R.)

**Keywords:** Crohn’s disease, magnetic resonance enterography (MRE), MaRIA score, diffusion-weighted imaging, DWI MaRIA, cluster analysis, disease activity assessment

## Abstract

**Background/Objectives:** Crohn’s disease (CD) requires precise, noninvasive monitoring to guide therapy and support treat-to-target management. Magnetic resonance enterography (MRE), particularly diffusion-weighted imaging (DWI), is the preferred cross-sectional technique for assessing small-bowel inflammation. Indices such as the Magnetic Resonance Index of Activity (MaRIA) and its diffusion-weighted variant (DWI MaRIA) are widely used for grading disease activity. This study evaluated whether unsupervised clustering of MRI-derived features can complement these indices by providing more coherent and biologically grounded stratification of disease activity. **Materials and Methods:** Fifty patients with histologically confirmed CD underwent 1.5 T MRE. Of 349 bowel segments, 84 were pathological and classified using literature-based thresholds (MaRIA, DWI MaRIA) and unsupervised clustering. Differences between inactive, active, and severe disease were analyzed using multivariate analysis of variance (MANOVA), analysis of variance (ANOVA), and *t*-tests. Mahalanobis distances were calculated to quantify and compare separation between categories. **Results:** Using MaRIA thresholds, 5, 16, and 63 segments were classified as inactive, active, and severe (Mahalanobis distances 2.60, 4.95, 4.12). Clustering redistributed them into 22, 37, and 25 (9.26, 24.22, 15.27). For DWI MaRIA, 21, 14, and 49 segments were identified under thresholds (3.59, 5.72, 2.85) versus 21, 37, and 26 with clustering (7.40, 16.35, 9.41). Wall thickness dominated cluster-derived separation, supported by diffusion metrics and the apparent diffusion coefficient (ADC). **Conclusions:** Cluster-derived classification yielded clearer and more biologically consistent separation of disease-activity groups than fixed thresholds, emphasizing its potential to refine boundary definition, enhance MRI-based assessment, and inform future AI-driven diagnostic modeling.

## 1. Introduction

Crohn’s disease (CD) is a chronic, relapsing inflammatory bowel disease (IBD) that can affect any segment of the gastrointestinal tract, most commonly the terminal ileum. Due to its transmural nature, CD is frequently associated with complications such as abscesses, fistulas, and strictures, resulting in significant morbidity and a lifelong need for disease monitoring [1,2]. The incidence of CD continues to rise worldwide, with a peak onset in adolescence and early adulthood [2]. Given its chronic course, patients with CD often undergo multiple imaging evaluations over their lifetime. In this context, magnetic resonance enterography (MRE) has become the modality of choice, offering detailed anatomical and functional information without ionizing radiation. This is particularly important in pediatric and young adult populations, where minimizing cumulative radiation exposure is a major concern [3,4]. Diffusion-weighted imaging (DWI), a functional magnetic resonance imaging (MRI) technique based on the Brownian motion of water molecules, has been integrated into MRE protocols as a non-contrast method for detecting inflammatory changes. Active inflammation is typically associated with restricted diffusion and reduced apparent diffusion coefficient (ADC) values, whereas chronic fibrotic segments tend to show higher ADC values than those observed in active inflammation [5,6]. Several studies have demonstrated that DWI can differentiate acute from chronic inflammation, with diagnostic performance comparable or superior to contrast-enhanced imaging [7,8]. Concerns regarding gadolinium-based contrast agents (GBCAs), including potential retention in brain tissue and risks in patients with impaired renal function, have further strengthened the role of DWI as a safer alternative for repeated evaluations [9,10]. To objectively assess disease activity, semiquantitative indices, such as the Magnetic Resonance Index of Activity (MaRIA) and the DWI-based MaRIA (also known as the Clermont score; DWI MaRIA), have been developed and validated [11,12]. The MaRIA index uses the mural thickness, edema, ulceration, and relative contrast enhancement, whereas DWI MaRIA is a non-contrast variant that substitutes the enhancement term with diffusion metrics (high-b DWI signal and ADC) and includes the T2 signal-intensity (SI) ratio per the Clermont formula [12,13]. Although these indices have demonstrated strong correlations with endoscopic findings, they rely on literature-based MaRIA cut-offs derived from heterogeneous populations and varied imaging protocols. In daily practice, this may lead to inconsistent results, particularly in patients with borderline or mild disease activity [12,13,14]. Advanced data analysis methods, such as cluster analysis, may offer a useful complementary approach to refine classification of bowel segment activity. Cluster analysis is a robust, unsupervised multivariate method widely applied in biomedical research to identify natural groupings within complex datasets [15]. In IBD, clustering techniques have been used to stratify patients by disease behavior, treatment response, and prognosis [16,17], yet their application to MRI-based activity indices remains largely unexplored. By grouping bowel segments according to intrinsic imaging patterns rather than rigid thresholds, cluster-derived classifications may provide more biologically coherent and clinically meaningful separations of disease activity. The aim of the present study was therefore to compare conventional literature-based MaRIA and DWI MaRIA classifications with cluster-derived groupings in order to evaluate which approach provides a more consistent and clinically relevant categorization of CD activity.

## 2. Materials and Methods

Fifty consecutive patients with histologically confirmed CD were enrolled. Referrals for MRE were based on clinical or laboratory indications, either during active disease or for therapy monitoring. All participants gave written informed consent. The protocol was approved by the institutional ethics committee and conducted in accordance with the Declaration of Helsinki.

### 2.1. Study Population

Fifty consecutive patients with histologically confirmed CD and under regular follow-up at the Clinic for Gastroenterology and Hepatology were enrolled. Patients were referred for MRE based on clinical or laboratory indications, either during active disease or for therapeutic monitoring. All participants provided written informed consent prior to imaging. The study was approved by the institutional ethics committee and conducted in accordance with the Declaration of Helsinki. Histologic confirmation of CD was obtained by endoscopic biopsy in all 50 patients, ensuring diagnostic verification prior to MRI analysis. Patients with prior bowel surgery were excluded because postoperative adhesions, anastomoses, and mesenteric remodeling can displace bowel loops across abdominal quadrants and distort native segment boundaries, potentially compromising the predefined seven-segment map and the comparability of segment-level measurements across the cohort. Characteristics of the study population are summarized in Table 1. Inclusion criteria comprised histologically verified CD, clinical indication for MRE as part of disease management, and successful completion of MRI examination with adequate image quality for interpretation. Exclusion criteria were prior surgical resection of the bowel, contraindications to MRI such as metallic implants, pacemakers, artificial heart valves, surgical clips, or insulin pumps, known allergy to gadolinium-based contrast agents (GBCAs), severe renal impairment defined as estimated glomerular filtration rate (eGFR) <30 mL/min, first trimester of pregnancy, and severe claustrophobia.

### 2.2. MR Acquisition

All examinations were performed with patients in the supine position. Imaging was carried out on a 1.5 Tesla scanner (Signa HDxT, GE Healthcare, Boston, MA, USA) using an eight-channel phased-array abdominal coil. To minimize peristaltic motion, 20 mg of intravenous hyoscine butylbromide (Buscopan) was administered immediately prior to scanning. For bowel distention, patients ingested 1500 mL of a biphasic oral contrast solution consisting of 500 mL mannitol and 1000 mL water over a 40 min period prior to imaging. The imaging protocol included coronal and axial T2-weighted Fast Imaging Employing Steady-state Acquisition (FIESTA; balanced steady-state free precession, bSSFP) sequences with and without fat suppression (repetition time/echo time, TR/TE = 3.9/1.6 ms; slice thickness 6.0 mm with 1.0 mm gap; field of view, FOV = 614 × 440 mm^2^ coronal/600 × 430 mm^2^ axial; acquisition matrix = 192 × 320; number of excitations, NEX = 1; flip angle = 75°). DWI was acquired axially with b = 0 and 800 s/mm^2^ (TR/TE = 8000.0/78.6 ms, NEX = 4), and coronally with b = 0 and 1400 s/mm^2^ (TR/TE = 2000.0/71.6 ms, NEX = 4), both with geometry identical to the corresponding T2 FIESTA planes. Axial three-dimensional (3D) Liver Acquisition with Volume Acceleration (LAVA) was performed during a single breath-hold (TR/TE = 4.1/2.1 ms; slice thickness = 4.4 mm; FOV = 614 × 440 mm^2^; matrix = 320 × 160; NEX = 0.70; flip angle = 12°). Coronal multiphase FIESTA was obtained with TR/TE = 3.9/1.7 ms; 15 acquisitions; slice thickness = 6.0 mm with 1.0 mm gap; FOV = 614 × 440 mm^2^; matrix = 192 × 320; NEX = 1; flip angle = 75°. Dynamic post-contrast imaging was performed with coronal multiphase 3D LAVA with fat suppression. Acquisition included one pre-contrast and four post-contrast phases (15 s, 45 s, 70 s, 90 s) (TR/TE = 4.3/2.1 ms; slice thickness = 4.4 mm; FOV = 600 × 430 mm^2^; matrix = 320 × 160; NEX = 0.73; flip angle = 12°). Gadobutrol (Gadovist, 0.1 mL/kg; 175.25 mg gadolinium per mL, Bayer AG, Leverkusen, Germany) was injected intravenously at 1.5–2.5 mL/s, followed by 20 mL saline at the same rate, using a power injector (Optistar LE; Liebel-Flarsheim Company LLC, Cincinnati, OH, USA). Axial and coronal post-contrast 3D LAVA sequences were acquired with identical parameters as the corresponding pre-contrast sequences. Coronal DWI, multiphase coronal FIESTA, and 3D LAVA were obtained with Array Spatial Sensitivity Encoding Technique (ASSET) parallel imaging (acceleration factor = 2.0). ADC maps were calculated using a mono-exponential model.

### 2.3. Image Analysis and Measurements

The gastrointestinal tract was divided into seven anatomical segments per patient: jejunum, proximal ileum, terminal ileum, cecum and ascending colon, transverse colon, descending colon, and sigmoid colon with rectum, yielding 350 segments in total. To standardize segment selection, segment labels were assigned as follows: segment I (jejunum) was assumed in the left upper quadrant of the abdomen; segment II (proximal and middle ileum) in the left lower quadrant; segment III (distal and terminal ileum) in the right upper and right lower quadrants; segment IV (cecum and ascending colon, right colon); segment V (transverse colon); segment VI (descending colon, left colon); and segment VII (sigmoid colon and rectum). This seven-segment scheme reflects a published convention and represents one of several accepted segmentations used in MRE studies [11,12,18,19], selected to provide fixed anatomic rules for reproducible segment labeling across patients. The perianal region was excluded. One poorly distended pathological segment was excluded, leaving 349 segments for analysis. Wall thickness was measured on T2-weighted fat-suppressed images. Edema was assessed by the signal intensity (SI) ratio between the bowel wall and the contralateral psoas muscle. Ulceration was evaluated qualitatively. The SI of the bowel wall was measured with regions of interest (ROIs) placed over the most affected area and normalized to the SI of the psoas muscle (T2 SI ratio). ADC values were measured on coronal DWI (b = 1400 s/mm^2^) at corresponding anatomical locations, with ROIs defined on T2-weighted reference images. ROI size was adapted to segmental thickness (0.2–1.0 cm^2^). Segments were considered morphologically normal if no thickening, edema, ulceration, or pathological contrast enhancement was present, and if not adjacent to inflamed bowel. Segmental status was confirmed in at least two planes and multiple sequences. Relative contrast enhancement (RCE) was calculated from pre- and post-contrast T1-weighted values according to the formula: RCE = [(wall signal intensity (WSI) post-gadolinium − WSI pre-gadolinium)/WSI pre-gadolinium] × 100 × (SD noise pre-gadolinium/SD noise post-gadolinium). Activity indices were calculated as follows:

MaRIA = 1.5 × wall thickness (mm) + 0.02 × RCE + 5 × edema + 10 × ulceration [11].

DWI MaRIA = 1.5 × wall thickness (mm) + 3.5 × DWI signal + 1.75 × T2 SI ratio − 1.321 × ADC × 10^3^ [12].

**Figure 1:**
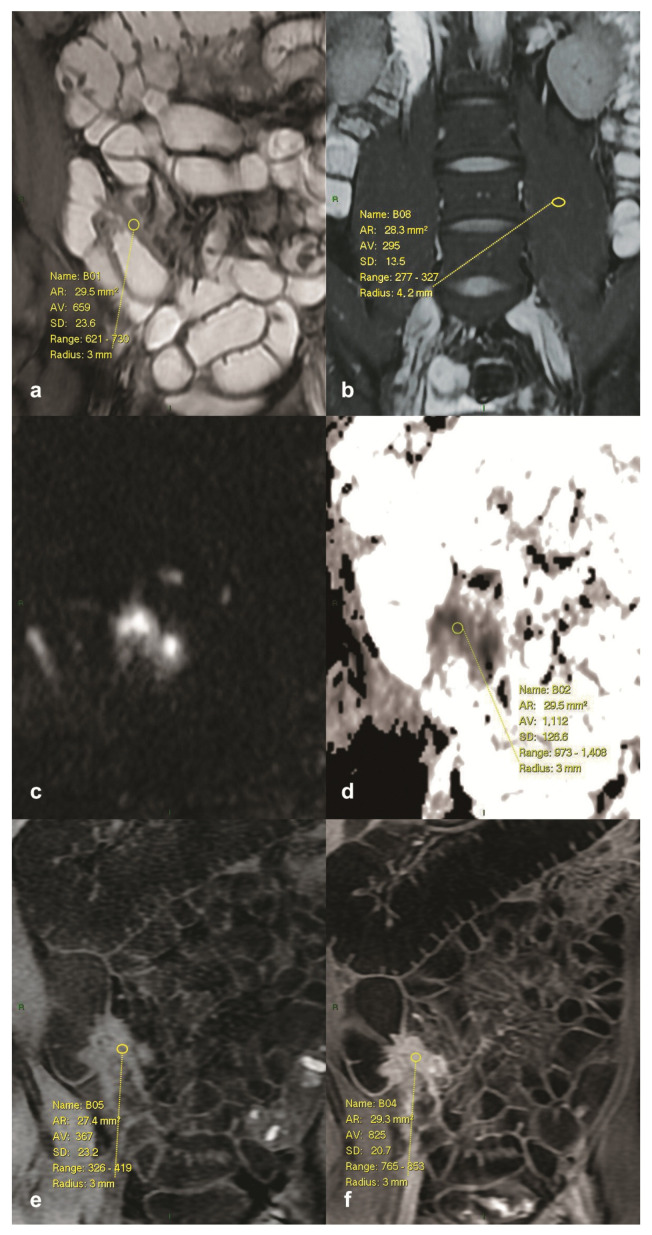
Multimodal magnetic resonance enterography (MRE) assessment of Crohn’s disease in segment III (distal and terminal ileum). Representative example of region-of-interest (ROI) placement and quantitative measurements across different magnetic resonance imaging (MRI) sequences. (**a**) Coronal T2-weighted image demonstrating mural thickening and hyperintense signal in the terminal ileum, with ROI positioned over the most affected segment for signal intensity (SI) measurement. (**b**) Corresponding coronal T2-weighted fat-suppressed (FS) image with ROI placed over the contralateral psoas muscle, serving as the reference structure for normalization of bowel-wall SI. (**c**) Coronal diffusion-weighted imaging (DWI; b = 1400 s/mm^2^) showing restricted diffusion in the same segment, consistent with active inflammation. (**d**) Apparent diffusion coefficient (ADC) map derived from DWI, illustrating reduced ADC values within the ROI. (**e**) Unenhanced coronal T1-weighted image of the terminal ileum with ROI placed to determine baseline bowel-wall SI. (**f**) Post-contrast coronal T1-weighted Liver Acquisition with Volume Acceleration (LAVA) image (70 s after gadobutrol administration), demonstrating marked mural enhancement within the ROI. Relative contrast enhancement (RCE) was derived from paired pre- and post-contrast measurements. These multiparametric evaluations formed the basis for calculating the Magnetic Resonance Index of Activity (MaRIA) and the diffusion weighted imaging based MaRIA (DWI MaRIA) activity indices.

Segments were stratified into three groups using literature-based MaRIA cut-offs: for MaRIA, ≤7 indicated inactive disease, >7 and ≤11 defined active disease, and >11 defined severe disease; for DWI MaRIA, ≤8 indicated inactive disease, >8 and ≤12.5 defined active disease, and >12.5 defined severe disease (Figure 1).

### 2.4. Statistical Analysis

Two complementary strategies were applied to classify bowel segments into disease activity groups. The first approach relied on literature-based MaRIA categorization using established cut-off values, while the second approach employed cluster analysis performed separately on the final composite indices (the MaRIA index and the DWI MaRIA index), in order to identify data-driven groupings. The number of clusters was prespecified as three, consistent with the expected clinical categories of inactive, active, and severe disease. Clustering was implemented separately on the final MaRIA index and on the final DWI MaRIA index after z-standardization, using one-dimensional (1D) Euclidean inter-segment distances and agglomerative hierarchical clustering with single-link (nearest-neighbor) linkage. Starting from singleton clusters, agglomeration proceeded until exactly three clusters remained (k = 3). We clustered on the composite indices rather than on individual index components to align directly with clinical cut-off usage. Separation patterns were inspected on the agglomeration schedule and dendrogram to assess chaining [20,21]. Group differences were tested for gender and age, with no significant differences observed. Normality of distribution was assessed using skewness, kurtosis, and *p*-values. Multivariate analysis of variance (MANOVA) was used to examine overall differences between the three disease-severity groups. When significant, one-way analysis of variance (ANOVA) was applied to individual parameters, followed by post hoc two-tailed Student’s *t*-tests to assess pairwise differences. For clarity of presentation, the results of *t*-tests are displayed within the same tables as discriminant analysis (DA). Superscript markers indicate significance levels: *^1^ denotes a significant difference compared to the lower-value group, and *^2^ indicates that the highest-value group differed significantly from both other groups. All comparisons were considered statistically significant at *p* < 0.05. DA was performed to determine which imaging parameters contributed most to group separation. The relative contribution of each parameter was expressed as a percentage, and group homogeneity was calculated to evaluate classification consistency. Pairwise Mahalanobis distances were computed to quantify the degree of separation between groups and to compare the effectiveness of literature-based MaRIA versus cluster-derived classifications (larger distances indicating clearer separation). Internal validation of the clustering, then assessed within-cluster homogeneity as a measure of cohesion and pairwise Mahalanobis distances between the three clusters as a measure of separation. All mathematical analyses were performed using SPSS software (version 29.0; IBM Corp., Armonk, NY, USA).

All aspects of this study, including design, data acquisition, statistical analysis, interpretation, and manuscript preparation, were conducted entirely by the authors and members of the research team without the use of generative artificial intelligence (GenAI) systems or large language models (LLMs). The authors take full responsibility for the integrity, accuracy, and originality of all data and analyses presented in this study. The work represents genuine human intellectual contribution and scientific judgment.

## 3. Results

Across 50 patients, 350 bowel segments were evaluated (seven per patient). After exclusion of one poorly distended pathological segment, 349 segments remained; of these, 84 met the predefined criteria for pathological involvement and entered the comparative analyses (literature-based cut-offs and data-driven clustering).

### 3.1. Literature-Based MaRIA Classification

Application of the literature-based MaRIA classification resulted in 5 segments being classified as inactive, 16 as active, and 63 as severe (Table 2, Table 3 and Table 4). The distribution of the examined MRI-derived variables within these groups was approximately normal, as indicated by descriptive statistics and dispersion parameters. MANOVA revealed significant overall differences between the three severity groups (*p* < 0.001). One-way ANOVA confirmed significant between-group differences for all examined variables (*p* < 0.001). Pairwise comparisons were assessed with two-tailed *t*-tests, with results incorporated into the discriminant-analysis table (Table 5 indicated by *^1^ and *^2^). Discriminant analysis identified DWI MaRIA, MaRIA, and ADC as the strongest contributors to intergroup differentiation, while confirming high within-group homogeneity (Table 5). Mahalanobis distances (Table 6) quantified the degree of separation between the three MaRIA literature-based groups, providing a multivariate measure of intergroup similarity and dissimilarity.

### 3.2. Literature-Based DWI MaRIA (Clermont) Classification

Using literature-based DWI MaRIA classification yielded 21 inactive, 14 active, and 49 severe segments. The distribution of the examined MRI-derived variables within these groups was approximately normal, as indicated by descriptive statistics and dispersion parameters (Table 7, Table 8 and Table 9). MANOVA revealed significant overall differences between the three groups (*p* < 0.001). One-way ANOVA confirmed significant between-group differences for all examined variables (*p* < 0.001). Post hoc pairwise differences were assessed using two-tailed *t*-tests, and the results are incorporated in the discriminant analysis table (Table 10, indicated by *^1^ and *^2^). DA highlighted DWI MaRIA, wall thickness, and ADC as the most influential contributors to group differentiation, with homogeneity exceeding 95% across groups (Table 10). Mahalanobis distances (Table 11) quantified the intergroup distances between the three DWI MaRIA literature-based groups.

### 3.3. Cluster-Derived MaRIA Classification

Unsupervised clustering of MaRIA-derived variables produced 22 inactive, 37 active, and 25 severe segments (Table 12, Table 13 and Table 14). The distribution of the examined MRI-derived variables within these clusters was approximately normal, as indicated by descriptive statistics and dispersion parameters. Importantly, the minimum and maximum values observed in the descriptive statistics for each cluster define new data-driven thresholds for group boundaries, representing cluster-derived cut-offs that more accurately reflect the characteristics of the analyzed cohort. MANOVA revealed significant differences between the three cluster-derived groups (*p* < 0.001). One-way ANOVA confirmed significant between-group differences for all examined variables (*p* < 0.001). Post hoc pairwise differences were assessed using two-tailed *t*-tests, and the results are incorporated in the discriminant analysis table (Table 15 indicated by *^1^ and *^2^). DA identified DWI MaRIA, MaRIA, and ADC as the three most important contributors to group differentiation, with high within-cluster homogeneity (Table 15). Mahalanobis distances were 9.26 for inactive vs. active, 24.22 for inactive vs. severe, and 15.27 for active vs. severe. Compared with literature-based MaRIA, the corresponding distances were larger under clustering (inactive vs. active: 9.26 vs. 2.60; inactive vs. severe: 24.22 vs. 4.95; active vs. severe: 15.27 vs. 4.12) (Table 16).

### 3.4. Cluster-Derived DWI MaRIA Classification

Clustering of DWI MaRIA-derived variables yielded 21 inactive, 37 active, and 26 severe segments (Table 17, Table 18 and Table 19). The distribution of the examined MRI-derived variables within these clusters was approximately normal, as indicated by descriptive statistics and dispersion parameters. The minimum and maximum values in the descriptive statistics provide new, cluster-derived thresholds for distinguishing between inactive, active, and severe disease. MANOVA revealed significant differences between the three cluster-derived groups (*p* < 0.001). One-way ANOVA confirmed significant between-group differences for all examined variables (*p* < 0.001). Post hoc pairwise differences were assessed using two-tailed *t*-tests, and the results are incorporated in the discriminant analysis tables (Table 20, indicated by *^1^ and *^2^). DA identified DWI MaRIA, wall thickness, and ADC as the most relevant contributors to group differentiation, with high internal homogeneity (Table 20). Mahalanobis distances were 7.40 for inactive vs. active, 16.35 for inactive vs. severe, and 9.41 for active vs. severe. Relative to the literature-based DWI MaRIA classification, these distances were larger under clustering (inactive vs. active: 7.40 vs. 3.59; inactive vs. severe: 16.35 vs. 5.72; active vs. severe: 9.41 vs. 2.85) (Table 21).

## 4. Discussion

This study explores whether data-driven grouping can improve MRI-based classification of Crohn’s disease (CD) activity beyond literature-based categories derived from fixed thresholds. In our analysis, bowel segments were classified both by applying literature-based thresholds from established indices (MaRIA and DWI MaRIA) and by using data-driven clustering. For each approach, we examined which parameters contributed most strongly to separating inactive, active, and severe segments. While the diffusion-based DWI MaRIA consistently showed the highest contribution under literature-based thresholds, clustering shifted the emphasis toward wall thickness, with DWI MaRIA and ADC remaining close behind. This yielded clearer separation of disease-activity groups and demonstrated that the two approaches reflect different aspects of the underlying biology. Our cluster-derived groups showed wider separation than literature-derived categories, and the observed differences were consistent with biologically expected imaging patterns in wall thickness, diffusion, and signal-intensity measures. In practical terms, clustering yielded boundaries that better reflected the expected gradients of inflammation across segments in this cohort, while remaining consistent with established MRI indices. MRE addresses a central limitation of ileocolonoscopy: the inability to evaluate the proximal small bowel comprehensively and to quantify transmural inflammation. Contemporary guidance from the European Crohn’s and Colitis Organisation (ECCO), the European Society of Gastrointestinal and Abdominal Radiology (ESGAR), the European Society of Pathology (ESP), and the International Bowel Ultrasound Group (IBUS) underscores the value of cross-sectional imaging for assessing mural and extramural disease, treatment response, and complications, particularly in situations where endoscopy cannot fully capture disease extent [22,23,24,25,26]. Recent reviews likewise emphasize MRE as a radiation-free modality suitable for repeated follow-up and for integrating objective, quantitative features into treat-to-target strategies [27,28,29,30]. CT enterography and intestinal ultrasound remain complementary cross-sectional techniques. While CT provides high spatial resolution and wide availability, MRE offers superior soft-tissue contrast, quantitative evaluation of transmural inflammation, and no ionizing radiation, making it preferable for repeated longitudinal monitoring. The data-driven clustering framework proposed here could also be explored in CT or ultrasound datasets in future comparative studies to assess whether similar cohort-specific boundaries emerge across modalities. We performed MRE at 1.5 T, a choice consistent with reports that, despite higher signal-to-noise ratio at 3 T, small-bowel imaging does not consistently achieve superior diagnostic performance at higher field strength and may be more prone to motion and susceptibility artifacts [30,31]. A notable limitation of the threshold-based MaRIA classification was that only five segments were categorized as inactive, despite the broader MRI profile suggesting a higher proportion of non-inflamed bowel. Unlike the predefined thresholds of the MaRIA and DWI MaRIA indices, clustering yielded new cohort-specific boundaries that redistributed segments into inactive, active, and severe groups in a way that was more consistent with the overall imaging profile. This discrepancy highlights how fixed cut-offs can fail to capture the distribution of segments, whereas cluster-derived groupings provided a more balanced and biologically consistent classification. Our findings offer a complementary perspective to recent efforts proposing simplified or pediatric-specific activity indices, such as the simplified MaRIA (sMaRIA), the Pediatric simplified MaRIA (P-sMaRIA), the Pediatric Inflammatory Crohn’s MRE Index (PICMI), and the modified Clermont score [32,33,34,35,36,37,38]. While those efforts recalibrate established formulas to improve clinical performance, our analysis shows that direct application of original literature-based thresholds can yield distributions that are poorly aligned with the broader imaging profile. In contrast, cluster-derived boundaries adapt to the characteristics of the cohort and generate classifications that are more consistent with biological expectations. The clinical relevance of redefining boundaries emerges clearly from our results, but these boundaries were derived from our own cohort and imaging protocol. Rather than suggesting universal cut-offs, our findings support deriving cohort-specific clusters at each center to refine classification in the local setting. Activity thresholds influence therapeutic decisions, including initiation or modification of treatment and the timing of response assessment. In practice, centers performing MRE for CD can use accumulated imaging data to establish cluster-derived reference ranges specific to their scanners and patient populations, supporting more consistent interpretation and treatment decisions. If cohort-specific clustering identifies groups that are more clearly separated, this could enhance classification accuracy at the margins and support more precise decision rules for treat-to-target care. Similar to the development of the original MaRIA and DWI MaRIA indices, this study was designed as a methodological proof of concept focused on improving the classification of disease activity rather than predicting treatment outcomes. The clustering approach refined the separation of inactive, active, and severe disease categories within the established MRI framework, without the intention to replace existing indices. Although therapeutic outcomes were not analyzed, future prospective studies should test whether cluster-derived categories can predict treatment escalation, surgery, or relapse-free survival, thereby linking imaging-derived clusters to clinical decision-making. These refinements are particularly relevant for the increasingly valued treatment targets of transmural response and healing, which reflect resolution of inflammation throughout the bowel wall rather than mucosa alone [39,40,41,42]. Cluster-derived boundaries can therefore serve as an initial framework for calibration at the level of individual MRI centers. They can be prospectively validated and, if shown to be robust, incorporated into routine reporting alongside widely used indices. At the same time, reproducibility across scanners and protocols remains essential, especially for multicenter studies. Standardized terminology and reporting frameworks promoted by international guidelines aim to reduce variability introduced by differences in technique [22,23,24,34]. Centers adopting cluster-derived thresholds should document protocol details, maintain rigorous quality control of ROI methodology and measurement procedures, and periodically re-estimate boundaries when hardware or sequences change. Multicenter consortia could use harmonized pipelines to test whether cluster-based boundaries stabilize when sample size and diversity increase [16,17]. Looking forward, radiomics and artificial intelligence (AI) offer a natural extension. Deep learning and conventional radiomics applied to MRE can capture patterns beyond human perception and show promise for distinguishing inflammation from fibrosis and predicting outcomes [43,44,45,46]. Incorporating cluster-derived labels in future AI studies may help define more biologically coherent phenotypes, but these applications will require rigorous external validation before translation into clinical practice. Cluster-derived categories can serve as a form of weak supervision, guiding AI models to recognize biologically meaningful distinctions in disease activity even when full histologic labeling is not available. In this approach, clusters function as pseudo-labels that orient feature learning toward patterns reflecting true inflammatory burden rather than predefined thresholds. Recent studies demonstrate that radiomic and deep learning models can extract quantitative MRI and CT features that distinguish inflammatory and fibrotic components of CD and predict the therapeutic response [47,48,49,50]. Weakly supervised and self-supervised learning strategies are increasingly used in medical imaging to address label scarcity and enhance generalizability across datasets [49]. These concepts support the potential of cluster-informed AI pipelines for more objective and reproducible disease assessment.

This study has several limitations. It was a single-center study conducted on a 1.5 T platform, which may limit the generalizability of the results. Future studies using 3 T systems could explore whether higher field strength further improves the sensitivity and reproducibility of cluster-derived boundaries [29,30]. Second, our reference standard relied solely on imaging, which inherently emphasizes mural inflammation and transmural features rather than mucosal status. Histologic grading across all bowel segments is not technically feasible, since endoscopic biopsy provides mucosal samples from a limited portion of the colon and terminal ileum, whereas MRE assesses transmural and proximal small-bowel inflammation beyond the reach of endoscopy. Consequently, imaging–histology correspondence is inherently partial, and MRI-based evaluation reflects a broader, transmural disease component rather than mucosal severity alone. Recent evidence further supports this concept, emphasizing that transmural healing has emerged as an independent therapeutic target alongside mucosal healing and is associated with improved long-term outcomes in CD [50,51]. While endoscopy is traditionally regarded as the reference for assessing mucosal disease activity, it cannot comprehensively evaluate the proximal small bowel or transmural involvement. As a result, imaging-based and endoscopy-based assessments may diverge, with MRE capturing deeper wall and extramural changes that are invisible to mucosal inspection [22,26]. In addition, recent work has highlighted areas of discordance between MRE and ileocolonoscopy for ileal strictures, underscoring the need to integrate modalities when defining decision rules [52]. Third, the sample size was modest, which may limit the stability of clustering results, and prospective validation is required to ensure that “inactive,” “active,” and “severe” categories correspond to meaningful differences in prognosis and treatment response. Fourth, the relative importance of individual parameters may vary with differences in imaging protocols or patient populations. Nevertheless, the ranking observed in this cohort provides meaningful insight into disease characterization and can stimulate further validation in broader settings. Finally, we did not perform external validation or a direct comparison between cluster-derived classifications and simplified indices (such as sMaRIA, P-sMaRIA, or the modified Clermont score) for specific clinical decision-making. Multicenter studies with harmonized acquisition protocols will be essential to confirm the generalizability of these findings. Such evaluations remain an important priority for future research. Despite these limitations, our findings provide proof-of-concept that data-driven clustering can generate biologically consistent classifications that complement established indices.

## 5. Conclusions

Cluster-derived groupings of bowel segments based on MRI features achieved clearer distinction of disease-activity categories than literature-based classifications, with wall thickness, diffusion-based metrics, and ADC emerging as the most informative contributors. These data-driven boundaries offer a framework for local recalibration of cut-offs, improving the alignment between imaging assessments and clinical decision-making. Looking ahead, multicenter external validation across vendors and protocols, prospective linkage to therapeutic outcomes and transmural targets, and integration with radiomics/AI pipelines remain essential priorities. This approach also aligns with a learning-health-system perspective, where centers iteratively refine imaging classifications to better mirror the biology observed in their own patient populations.

## Figures and Tables

**Table 1:** Characteristics of the 50 Crohn’s disease patients. Data are presented as *n* (%), where *n* denotes the number of patients. CDAI = Crohn’s Disease Activity Index; MRE = magnetic resonance enterography.

Characteristic	Value
Female gender	22 (44%)
Male gender	28 (56%)
Median age of the patients	31.82 (18–66)
Previous intestinal resection	0
Abdominal pain	17 (34%)
Joint pain	5 (10%)
Vision problems	1 (2%)
Skin changes	2 (4%)
Active perianal fistulas	4 (8%)
Chronic perianal fistulas	3 (6%)
Temperature above 37.8 °C	5 (10%)
Loose stools	12 (24%)
Clinical activity (CDAI > 150)	35 (70%)
Initial MRE examination	15 (30%)
Monitoring of disease	35 (70%)
Suspicion of extraintestinal complications	7 (14%)
One pathological segment	23 (46%)
Two pathological segments	13 (26%)
Three or more pathological segments	14 (28%)
Comb sign	40 (80%)
Free intraperitoneal fluid	24 (48%)
Ulcerations of the bowel wall	22 (44%)
Increased density of locoregional fat	22 (44%)
Entero-enteral fistulas	4 (8%)
Abscess	1 (2%)

**Table 2:** Descriptive statistics and tests of normality for the inactive disease group according to the MaRIA literature-based classification (5).

Parameter	Mean	SD	Min	Max	CV	CI	Skewness	Kurtosis	*p*-Value
T2 SI wall/T2 SI m. psoas	1.28	0.27	1.1	1.8	21.25	0.94–1.62	1.44	0.17	0.321
Wall thickness (mm)	3.38	0.37	3.1	4.0	10.95	2.92–3.84	1.07	−0.33	0.639
MaRIA	6.27	0.36	5.8	6.7	5.75	5.82–6.71	−0.14	−1.54	0.999
DWI MaRIA	3.38	0.75	2.7	4.5	22.09	2.40–4.35	0.72	−0.93	0.869
ADC	1.66	0.26	1.4	2.1	15.52	1.34–1.98	1.21	0.10	0.656

SD—standard deviation; Min—minimum; Max—maximum; CV—coefficient of variation; CI—confidence interval.

**Table 3:** Descriptive statistics and tests of normality for the active disease group according to the MaRIA literature-based classification (16).

Parameter	Mean	SD	Min	Max	CV	CI	Skewness	Kurtosis	*p*-Value
T2 SI wall/T2 SI m. psoas	1.27	0.37	0.8	2.2	29.06	1.08–1.47	0.89	0.50	0.850
Wall thickness (mm)	3.93	0.71	3.1	5.4	18.08	3.55–4.31	0.91	−0.27	0.698
MaRIA	8.58	1.13	7.1	10.9	13.18	7.98–9.18	0.30	−0.74	0.941
DWI MaRIA	4.65	1.21	3.2	6.9	25.97	4.01–5.30	0.88	−0.35	0.508
ADC	1.39	0.16	1.0	1.7	11.55	1.31–1.48	−0.21	0.53	0.795

**Table 4:** Descriptive statistics and tests of normality for the severe disease group according to the MaRIA literature-based classification (63).

Parameter	Mean	SD	Min	Max	CV	CI	Skewness	Kurtosis	*p*-Value
T2 SI wall/T2 SI m. psoas	1.89	0.44	1.1	3.0	23.58	1.77–2.00	0.44	−0.34	0.556
Wall thickness (mm)	5.68	1.20	3.5	8.8	21.17	5.38–5.99	0.46	0.09	0.927
MaRIA	20.11	6.05	11.4	33.7	30.08	18.58–21.63	0.44	−1.26	0.019
DWI MaRIA	16.77	4.99	9.9	27.1	29.75	15.51–18.03	0.37	−1.24	0.063
ADC	1.13	0.10	0.9	1.4	8.79	1.11–1.16	0.50	−0.07	0.310

**Table 5:** Characteristics and homogeneity for the MaRIA literature-based groups in relation to MRI-derived parameters, including relative contributions of parameters and pairwise *t*-test results. *^1^ indicates a significant difference compared to the group with lower values, while *^2^ indicates that the group with the highest value differed significantly from both other groups.

	Active	Inactive	Severe Disease	Contribution %
DWI MaRIA	Lower	Moderate *^1^	Higher *^2^	38.047
MaRIA	Lower	Moderate *^1^	Higher *^2^	28.282
ADC	Higher *^2^	Moderate *^1^	Lower	22.023
Wall thickness	Lower	Moderate	Higher *^2^	8.954
T2 SI wall/T2 SI m. psoas	Moderate	Lower	Higher *^2^	2.695
n/m	5/5	16/16	61/63	
%	100.00	100.00	96.83	

**Table 6:** Mahalanobis distances between groups in the MaRIA literature-based classification.

	Inactive	Active	Severe Disease
Inactive	0.00	2.60	4.95
Active	2.60	0.00	4.12
Severe disease	4.95	4.12	0.00

**Table 7:** Descriptive statistics and tests of normality for the inactive disease group according to the DWI MaRIA literature-based classification (21).

Parameter	Mean	SD	Min	Max	CV	CI	Skewness	Kurtosis	*p*-Value
T2 SI wall/T2 SI m. psoas	1.27	0.34	0.8	2.2	26.89	1.12–1.43	0.96	0.72	0.275
Wall thickness (mm)	3.80	0.68	3.1	5.4	17.93	3.49–4.11	1.12	0.32	0.732
MaRIA	8.03	1.42	5.8	10.9	17.64	7.39–8.67	0.19	−0.90	0.799
DWI MaRIA	4.35	1.23	2.7	6.9	28.32	3.79–4.91	0.91	0.06	0.691
ADC	1.46	0.22	1.0	2.1	14.73	1.36–1.55	0.96	2.43	0.608

**Table 8:** Descriptive statistics and tests of normality for the active disease group according to the DWI MaRIA literature-based classification (14).

Parameter	Mean	SD	Min	Max	CV	CI	Skewness	Kurtosis	*p*-Value
T2 SI wall/T2 SI m. psoas	1.55	0.44	1.1	2.6	28.71	1.29–1.81	0.94	0.02	0.544
Wall thickness (mm)	4.24	0.44	3.5	4.9	10.28	3.99–4.49	−0.09	−0.99	0.996
MaRIA	13.76	0.88	11.4	15.1	6.41	13.25–14.27	−1.14	1.71	0.993
DWI MaRIA	11.01	0.72	9.2	12.1	6.51	10.60–11.43	0.01	−1.19	0.896
ADC	1.20	0.09	1.1	1.4	7.16	1.15–1.25	0.33	−1.18	0.895

**Table 9:** Descriptive statistics and tests of normality for the severe disease group according to the DWI MaRIA literature-based classification (49).

Parameter	Mean	SD	Min	Max	CV	CI	Skewness	Kurtosis	*p*-Value
T2 SI wall/T2 SI m. psoas	1.98	0.40	1.4	3.0	20.15	1.87–2.10	0.72	−0.38	0.465
Wall thickness (mm)	6.10	1.02	4.1	8.8	16.73	5.80–6.39	0.74	0.71	0.606
MaRIA	21.92	5.65	13.7	33.7	25.79	20.30–23.55	1.13	−1.37	0.174
DWI MaRIA	18.41	4.43	12.6	27.1	24.04	17.14–19.68	0.19	−1.37	0.095
ADC	1.11	0.10	0.9	1.4	8.61	1.09–1.14	0.73	0.60	0.396

**Table 10:** Characteristics and homogeneity for the DWI MaRIA literature-based groups in relation to MRI-derived parameters, including relative contributions of parameters and pairwise *t*-test results. *^1^ indicates a significant difference compared to the group with lower values, while *^2^ indicates that the group with the highest value differed significantly from both other groups.

	Inactive	Active	Severe Disease	Contribution %
DWI MaRIA	Lower	Moderate *^1^	Higher *^2^	41.499
MaRIA	Lower	Moderate *^1^	Higher *^2^	26.636
ADC	Higher *^2^	Moderate *^1^	Lower	13.730
T2 SI wall/T2 SI m. psoas	Lower	Moderate *^1^	Higher *^2^	13.524
Wall thickness	Lower	Moderate *^1^	Higher *^2^	4.611
n/m	21/21	12/14	48/49	
Homogeneity %	100	85.71	97.96	

**Table 11:** Mahalanobis distances between groups in the DWI MaRIA literature-based classification.

	Inactive	Active	Severe Disease
Inactive	0.00	3.59	5.72
Active	3.59	0.00	2.85
Severe disease	5.72	2.85	0.00

**Table 12:** Descriptive statistics and tests of normality for the inactive disease group according to the MaRIA cluster-derived classification (22).

Parameter	Mean	SD	Min	Max	CV	CI	Skewness	Kurtosis	*p*-Value
T2 SI wall/T2 SI m. psoas	1.27	0.34	0.8	2.2	26.31	1.12–1.42	1.00	0.92	0.263
Wall thickness (mm)	3.81	0.67	3.1	5.4	17.51	3.52–4.11	1.07	0.33	0.688
MaRIA	8.18	1.56	5.8	11.4	19.07	7.49–8.88	0.33	−0.72	0.924
DWI MaRIA	4.64	1.81	2.7	10.7	38.98	3.83–5.44	1.88	0.76	0.075
ADC	1.45	0.21	1.0	2.1	14.72	1.35–1.54	1.02	2.49	0.477

**Table 13:** Descriptive statistics and tests of normality for the active disease group according to the MaRIA cluster-derived classification (37).

Parameter	Mean	SD	Min	Max	CV	CI	Skewness	Kurtosis	*p*-Value
T2 SI wall/T2 SI m. psoas	1.81	0.44	1.1	2.7	24.17	1.67–1.96	0.34	−0.68	0.508
Wall thickness (mm)	5.49	1.15	3.5	8.4	20.95	5.10–5.87	0.25	−0.38	0.997
MaRIA	15.66	1.90	13.1	20.0	12.13	15.03–16.30	0.63	−0.54	0.734
DWI MaRIA	13.14	1.92	9.9	17.9	14.62	12.50–13.78	0.18	−0.51	0.955
ADC	1.14	0.09	0.9	1.4	8.15	1.11–1.17	0.15	0.06	0.505

**Table 14:** Descriptive statistics and tests of normality for the severe disease group according to the MaRIA cluster-derived classification (25).

Parameter	Mean	SD	Min	Max	CV	CI	Skewness	Kurtosis	*p*-Value
T2 SI wall/T2 SI m. psoas	2.03	0.42	1.4	3.0	20.77	1.85–2.20	0.81	−0.26	0.303
Wall thickness (mm)	6.04	1.21	4.1	8.8	20.02	5.54–6.54	0.72	0.21	0.835
MaRIA	27.04	2.35	23.9	33.7	8.68	26.07–28.01	1.16	1.29	0.427
DWI MaRIA	22.38	2.04	18.9	27.1	9.12	21.54–23.22	0.16	0.24	0.713
ADC	1.12	0.11	1.0	1.4	9.69	1.08–1.17	0.98	0.19	0.311

**Table 15:** Characteristics and homogeneity for the MaRIA cluster-derived groups in relation to MRI-derived parameters, including relative contributions of parameters and pairwise *t*-test results. *^1^ indicates a significant difference compared to the group with lower values, while *^2^ indicates that the group with the highest value differed significantly from both other groups.

	Active	Inactive	Severe Disease	Contribution %
Wall thickness	Lower	Moderate *^1^	Higher *^2^	49.248
DWI MaRIA	Lower	Moderate *^1^	Higher *^2^	35.993
MaRIA	Lower	Moderate *^1^	Higher *^2^	8.133
ADC	Higher ^*2^	Moderate *^1^	Lower	6.009
T2 SI wall/T2 SI m. psoas	Lower	Moderate *^1^	Higher *^2^	0.617
n/m	21/22	31/37	25/25	
Homogeneity %	95.45	83.78	100	

**Table 16:** Mahalanobis distances between groups in the MaRIA cluster-derived classification.

	Inactive	Active	Severe Disease
Inactive	0.00	9.26	24.22
Active	9.26	0.00	15.27
Severe disease	24.22	15.27	0.00

**Table 17:** Descriptive statistics and tests of normality for the inactive disease group according to the DWI MaRIA cluster-derived classification (21).

Parameter	Mean	SD	Min	Max	CV	CI	Skewness	Kurtosis	*p*-Value
T2 SI wall/T2 SI m. psoas	1.27	0.34	0.8	2.2	26.89	1.12–1.43	0.96	0.72	0.275
Wall thickness (mm)	3.80	0.68	3.1	5.4	17.93	3.49–4.11	1.12	0.32	0.732
MaRIA	8.03	1.42	5.8	10.9	17.64	7.39–8.67	−0.19	−0.90	0.799
DWI MaRIA	4.35	1.23	2.7	6.9	28.32	3.79–4.91	0.91	0.06	0.691
ADC	1.46	0.22	1.0	2.1	14.73	1.36–1.55	0.96	2.43	0.608

**Table 18:** Descriptive statistics and tests of normality for the active disease group according to the DWI MaRIA cluster-derived classification (37).

Parameter	Mean	SD	Min	Max	CV	CI	Skewness	Kurtosis	*p*-Value
T2 SI wall/T2 SI m. psoas	1.80	0.45	1.1	2.7	24.92	1.65–1.95	0.34	−0.77	0.658
Wall thickness (mm)	5.37	1.06	3.5	7.4	19.75	5.02–5.72	0.01	−1.00	0.509
MaRIA	15.43	1.88	11.4	19.5	12.17	14.80–16.06	0.36	−0.42	0.509
DWI MaRIA	12.95	1.79	9.9	16.4	13.83	12.35–13.55	−0.04	−0.75	0.509
ADC	1.14	0.09	0.9	1.4	8.33	1.11–1.17	0.15	−0.12	0.683

**Table 19:** Descriptive statistics and tests of normality for the severe disease group according to the DWI MaRIA cluster-derived classification (26).

Parameter	Mean	SD	Min	Max	CV	CI	Skewness	Kurtosis	*p*-Value
T2 SI wall/T2 SI m. psoas	2.01	0.42	1.4	3.0	20.72	1.84–2.18	0.87	−0.17	0.330
Wall thickness (mm)	6.13	1.27	4.1	8.8	20.75	5.62–6.64	0.64	−0.21	0.809
MaRIA	26.77	2.68	20.0	33.7	10.26	25.68–27.85	0.32	1.45	0.681
DWI MaRIA	22.21	2.19	17.9	27.1	9.85	21.23–23.09	0.40	0.20	0.833
ADC	1.13	0.11	1.0	1.4	9.55	1.08–1.17	0.91	0.13	0.412

**Table 20:** Characteristics and homogeneity for the DWI MaRIA cluster-derived groups in relation to MRI-derived parameters, including relative contributions of parameters and pairwise *t*-test results. *^1^ indicates a significant difference compared to the group with lower values, while *^2^ indicates that the group with the highest value differed significantly from both other groups.

	Inactive	Active	Severe Disease	Contribution %
Wall thickness	Lower	Moderate *^1^	Higher *^2^	48.544
DWI MaRIA	Lower	Moderate *^1^	Higher *^2^	44.890
ADC	Higher *^2^	Moderate *^1^	Lower	4.262
MaRIA	Lower	Moderate *^1^	Higher *^2^	2.152
T2 SI wall/T2 SI m. psoas	Lower	Moderate *^1^	Higher *^2^	0.152
n/m	21/21	31/37	25/26	
homogeneity %	100	83.78	96.15	

**Table 21:** Mahalanobis distances between groups in the DWI MaRIA cluster-derived classification.

	Inactive	Active	Severe Disease
Inactive	0.00	7.40	16.35
Active	7.40	0.00	9.41
Severe disease	16.35	9.41	0.00

## Data Availability

The human data supporting the findings of this study are not publicly available due to ethical considerations, including participant privacy and data sensitivity. However, the data may be provided by the corresponding author upon request, subject to appropriate institutional and ethical approvals.

## Abbreviations

The following abbreviations are used in this manuscript:

ADC	Apparent Diffusion Coefficient
AI	Artificial Intelligence
ANOVA	Analysis of Variance
ASSET	Array Spatial Sensitivity Encoding Technique
CDAI	Crohn’s Disease Activity Index
CD	Crohn’s Disease
CI	Confidence Interval
CV	Coefficient of Variation
DA	Discriminant Analysis
DWI	Diffusion-Weighted Imaging
ECCO	European Crohn’s and Colitis Organisation
ESGAR	European Society of Gastrointestinal and Abdominal Radiology
ESP	European Society of Pathology
FIESTA	Fast Imaging Employing Steady-State Acquisition
FOV	Field of View
GBCA	Gadolinium-Based Contrast Agent
IBD	Inflammatory Bowel Disease
IBUS	International Bowel Ultrasound Group
LAVA	Liver Acquisition with Volume Acceleration
MaRIA	Magnetic Resonance Index of Activity
MANOVA	Multivariate Analysis of Variance
MRE	Magnetic Resonance Enterography
NEX	Number of Excitations
PICMI	Pediatric Inflammatory Crohn’s MRE Index
P-sMARIA	Pediatric simplified Magnetic Resonance Index of Activity
RCE	Relative Contrast Enhancement
ROI	Region of Interest
SD	Standard Deviation
SI	Signal Intensity
sMARIA	Simplified Magnetic Resonance Index of Activity
SPSS	Statistical Package for the Social Sciences
TE	Echo Time
TR	Repetition Time
WSI	Wall Signal Intensity
